# Genotype imputation in human genomic studies

**DOI:** 10.18699/vjgb-24-70

**Published:** 2024-10

**Authors:** A.A. Berdnikova, I.V. Zorkoltseva, Y.A. Tsepilov, E.E. Elgaeva

**Affiliations:** Institute of Cytology and Genetics of the Siberian Branch of the Russian Academy of Sciences, Novosibirsk, Russia Novosibirsk State University, Novosibirsk, Russia; Institute of Cytology and Genetics of the Siberian Branch of the Russian Academy of Sciences, Novosibirsk, Russia; Institute of Cytology and Genetics of the Siberian Branch of the Russian Academy of Sciences, Novosibirsk, Russia; Institute of Cytology and Genetics of the Siberian Branch of the Russian Academy of Sciences, Novosibirsk, Russia Novosibirsk State University, Novosibirsk, Russia

**Keywords:** imputation, genotyping, sequencing, genome-wide association study, human, DNA-microarray, импутация, генотипирование, секвенирование, полногеномный анализ ассоциаций, человек, ДНК-микрочип

## Abstract

Imputation is a method that supplies missing information about genetic variants that could not be directly genotyped with DNA microarrays or low-coverage sequencing. Imputation plays a critical role in genome-wide association studies (GWAS). It leads to a significant increase in the number of studied variants, which improves the resolution of the method and enhances the comparability of data obtained in different cohorts and/or by using different technologies, which is important for conducting meta-analyses. When performing imputation, genotype information from the study sample, in which only part of the genetic variants are known, is complemented using the standard (reference) sample, which has more complete genotype data (most often the results of whole-genome sequencing). Imputation has become an integral part of human genomic research due to the benefits it provides and the increasing availability of imputation tools and reference sample data. This review focuses on imputation in human genomic research. The first section of the review provides a description of technologies for obtaining information about human genotypes and characteristics of these types of data. The second section describes the imputation methodology, lists the stages of its implementation and the corresponding programs, provides a description of the most popular reference panels and methods for assessing the quality of imputation. The review concludes with examples of the use of imputation in genomic studies of samples from Russia. This review shows the importance of imputation, provides information on how to carry it out, and systematizes the results of its application using Russian samples

## Abstract (ru)

Импутация – это метод, позволяющий восстанавливать недостающую информацию о генетических вариантах, которые не удалось генотипировать напрямую с помощью ДНК-микрочипов или секвенирования с низким покрытием. Импутация играет важнейшую роль в полногеномном анализе ассоциаций (genome wide associations study, GWAS). Она приводит к существенному увеличению количества изучаемых вариантов, что повышает разрешающую способность метода и увеличивает сопоставимость данных, полученных в разных когортах и/или с помощью разных технологий, что важно при проведении метаанализов. При ее выполнении информацию о генотипах в исследуемой выборке, у которой известна только часть генетических вариантов, дополняют за счет эталонной (референсной) выборки, имеющей более полные данные о генотипах (чаще всего это результаты полногеномного секвенирования). Импутация стала неотъемлемой частью геномных исследований человека благодаря преимуществам, которые она дает, а также увеличению доступности инструментов для импутации и данных референсных выборок. Обзор посвящен импутации в геномных исследованиях человека. В первом разделе приводятся описание технологий получения информации о генотипах человека и характеристика получаемых типов данных. Во втором разделе представлена методология импутации, перечисляются этапы ее проведения и соответствующие программы, дается описание наиболее популярных референсных панелей и способов оценки качества импутации. В заключении представлены примеры использования импутации в геномных исследованиях выборок из России. Настоящий обзор показывает важность проведения импутации, дает информацию о том, как ее выполнять, и систематизирует результаты ее применения на примере российских выборок.

## Technologies for obtaining human genotype data and their features

Human genotype data are a key aspect for many genetic studies. There are several technologies developed to read, analyze and interpret genetic information. The most commonly used methods include Sanger sequencing, next generation sequencing (NGS), and DNA microarrays.

Genotyping using DNA microarrays

**Fig. 1.**
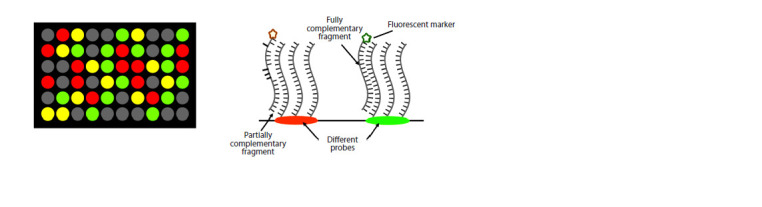
DNA microarray. Pseudocolor (red, yellow or green) is determined by the number of molecules bound to the probe and labeled with different dyes. For further explanation of the figure, see the text below.

A DNA microarray (or simply a “microchip” or “chip”, not to be confused with an RNA microarray, which is a different technology) is a small glass or silicon substrate, to which tens of thousands of probes (short single-stranded DNA fragments complementary to certain nucleotide sequences) are attached. These probes are arranged on the chip in such a way that each fragment can be identified by its location (Fig. 1).

During the analysis, fluorescent markers are attached to the studied DNA molecules, which were cut into fragments by restriction endonucleases, and placed on the chip. The target DNA fragments are bound to complementary DNA probes, and all remaining fragments are removed from the chip. Laser beams and computer processing are used to detect the fluorescence of fragments, record the emission (radiation) patterns and subsequently identify the sequences. This method is very fast and allows to simultaneously determine the nucleotide sequence of several DNA fragments (Govindarajan et al., 2012).

An alternative approach to solving the problem of genotyping was implemented by academician A.D. Mirzabekov in domestic developments to create gel microchips (Mirzabekov, 2003). They are a substrate made of glass, plastic or silicone with hemispherical drops of hydrogel fixed on its surface. The distinction of this method is that DNA fragments are immobilized in three-dimensional space, which provides greater sensitivity and capacity of the microchip. This technology has also found its application in RNA analysis, protein and cell biochips.

**Fig. 2.**
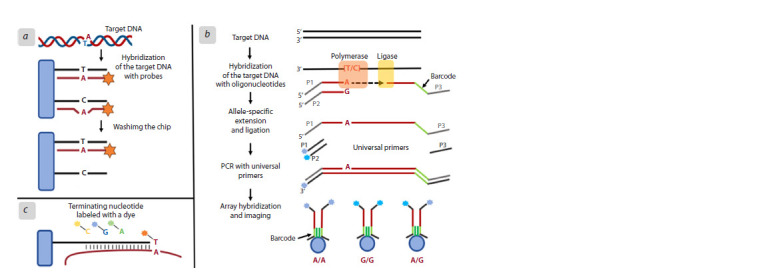
SNP detection strategies for DNA microarrays а – allele-specific hybridization; b – “Golden Gate” analysis by the Illumina company; c – arrayed primer extension.

There are several strategies for identifying single nucleotide polymorphism (SNP) for microarrays (Fig. 2).

Allele distinction by hybridization (Fig. 2a). The labeled target DNA hybridizes with probes containing a polymorphic site in the center. Correctly paired oligonucleotides are more stable (have a higher melting temperature) compared to duplexes with a non-complementary base. Therefore, after washing the chip under harsh temperature conditions, only correctly paired chains remain on it. It is common to use multiple fragments for each allele to improve the quality of the signal relative to noise (Wang D.G. et al., 1998).

“Golden Gate” analysis by the Illumina company (Fig. 2b). Two allele-specific oligonucleotides, each of which has a 5′ end with different universal primers (P1 and P2) (the primers are labeled with a unique fluorophore for subsequent site discrimination), hybridize in solution with genomic DNA. The third oligonucleotide, in addition to the universal primer (P3), has a tail with a “barcode” sequence complementary to the fragment on the chip. The allele-specific primers extended by a polymerase are ligated to a third oligonucleotide, after which the resulting fragments are amplified using the polymerase chain reaction and hybridized onto the chip. The use of multiple barcodes (one for each locus of interest) allows for analysis of several genomic regions at once (Fan et al., 2003).

Arrayed Primer Extension (APEX, Fig. 2c). Here, the chip contains a DNA fragment, the 5′ end of which is fixed to the substrate, and the 3′ end finishes with the nucleotide preceding the SNP being detected. Fragments of genomic DNA are hybridized to the chip, while the desired SNP remains unpaired. During the sequencing reaction, the nucleotide sequence attached to the substrate is extended by one terminating nucleotide labeled with a dye (Kurg et al., 2000). This nucleotide prevents further growth of the DNA chain, and the color of its dye allows you to determine which of the nucleotides (A, T, G or C) is located at the given position.

One of the main advantages of DNA microarrays is their high throughput capability (Hayat, 2002; Brown et al., 2024). The microarray provides the basis for simultaneous genotyping of thousands of different loci and detection of single nucleotide substitutions. Thus, microarrays are used to analyze large samples in order to genotype frequently occurring genetic variants (with a minor allele frequency in the population > 0.01).

However, there are some limitations in interpreting the results. Microarray data are typically binary (indicating the presence or absence of a specific allele), high-throughput (allowing the analysis of thousands or millions of SNPs), and requiring specialized analysis techniques to extract meaningful information. In this case, we are talking about software (for example, GenomeStudio (Illumina Inc., San Diego, CA, США)), which includes tools for quality control, genotype identification, visualization and data analysis. In addition, microchips can produce both false positive and false negative results. These issues highlight the importance of careful data interpretation and the need to use appropriate statistical methods to control quality and validate results.

## Genome sequencing

This chapter describes various sequencing technologies. Around 1976, two methods that could read hundreds of bases in half a day were developed – Sanger and Coulson strand termination and Maxam and Gilbert chemical cleavage (Maxam, Gilbert, 1977; Sanger et al., 1977). In both methods, the analyzed DNA is placed into four test tubes with different compositions of the reaction mixture for a specific type of nitrogenous base (A, T, G or C). Gilbert’s method uses DNA, radioactively labeled at one end, and a mixture of enzymes that specifically cut it before a certain type of nucleotide. Sanger sequencing, in contrast, involves primers and dideoxynucleotides that stop chain synthesis when radiolabeled dideoxynucleoside triphosphate (ddNTP; different in each tube) is included. Hence, as a result of implementing either method, labeled DNA fragments of different lengths that end with the same base are formed in each tube. Sequences are separated by length using polyacrylamide plate gel electrophoresis (one lane per base type) at single nucleotide resolution. The image obtained on X-ray film after electrophoresis allows researchers to restore the original sequence. The described methods immediately came into use, and by 1987, automated fluorescent Sanger sequencers could read about 1,000 bases per day (Smith et al., 1986; Connell et al., 1987).

In 2005, next generation sequencing (NGS) technologies were first introduced, which are based on two approaches. The first of these is sequencing by hybridization (SBH). The essence of the method is as follows: first, short sections of DNA are fixed on a glass substrate (DNA chip). Then the fragments to be identified are labeled with fluorophore and applied to the chip for hybridization with the fixed areas. Single-stranded DNA is washed away, and the hybridization pattern is read from the color marks and their brightness. An alternative approach in NGS is sequencing by synthesis (SBS) (Shendure et al., 2017).

As a rule, in technologies that use the SBS technique, prefragmented sequences are fixed in a flow cell, where cyclic synthesis of a new chain occurs. By sequentially adding one of the four deoxynucleotides to the mixture, having removed the previous ones in advance, it is possible to read signals from the cells where the synthesis reaction was successful. Therefore, the output provides information about where which nucleotide is located

**Fig. 3.**
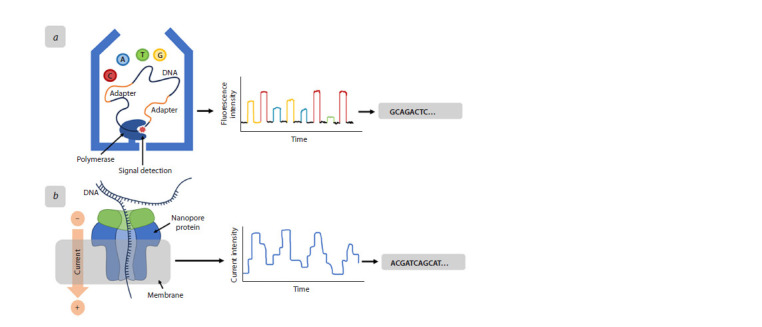
Third generation sequencing а – Pacific Biosciences; б – Oxford Nanopore Technology. See the text below for explanation.

Sequencing technologies with an approach other than NGS were first described in 2008–2009 and named “third generation sequencing” (Check Hayden, 2009). They include two main approaches (Fig. 3).

The first technology, Pacific Biosciences (PacBio) (Rhoads, Au, 2015), is designed to optically monitor DNA synthesis using a polymerase in real time. The structure has a hole less than half the light wavelength that limits fluorescent excitation to a small volume containing only the polymerase and its template (Fig. 3a). With such a device, only fluorescently labeled nucleotides included in the growing DNA strand emit signals of sufficient duration to be read. The error rate in this sequencing method is very high (about 10 %), but the errors are distributed randomly. With long reads and tolerance for high GC content and random errors, PacBio provides de novo assemblies of unprecedented quality in terms of accuracy and continuity.

The second major third-generation sequencing technology is Oxford Nanopore (ONT) (Deamer et al., 2016). This technique was first proposed in the 1980s. The special chamber where the sequencing process takes place is filled with an electrolytic solution and divided by a two-layer membrane with a nanopore (its dimensions are in the nanorange). Once voltage is applied, the electrolyte ions and the DNA molecule begin to move through the pore. Nucleic acid physically interferes with the migration of ions, which leads to fluctuations in current strength, which allows the nucleotide sequence to be determined (Fig. 3b). The main difference from other sequencing technologies is the extreme portability of nanopore devices, which can be as small as a memory stick (USB), as they rely on detecting electronic rather than optical signals.

## Comparison of technologies and their application to solve different problems

Most often, Illumina NGS technologies are used for largescale projects (whole-genome sequencing, transcriptome analysis and epigenetic profiling), but PacBio is more useful for de novo assembly, and ONT is more applicable for portable sequencing. The Sanger method is suitable for sequencing short DNA fragments such as individual genes, plasmids or viral genomes.

Also worth mentioning is a sequencing technology competing with Illumina, developed by Complete Genomics and MGI Tech, DNBSEQ-T7 (formerly known as MGISEQ-T7). In DNBSEQ-T7, the clonal amplification process occurs as a rolling circle, i. e., always from the original template, which eliminates the accumulation of DNA polymerase errors (Drmanac et al., 2010). The main advantages of MGI include lower cost compared to Illumina and the ability to process a larger volume of samples in a shorter time. As recent studies show, the new MGISEQ-2000 sequencer can be used as a fullfledged alternative to Illumina sequencers when conducting whole-genome studies (search for variants, identification of indels), the differences between the two platforms are insignificant (Korostin et al., 2020; Jeon et al., 2021; Feng et al., 2024).

Recently, the effectiveness of using whole-genome sequencing (WGS) for GWAS has been demonstrated (DePristo et al., 2011; Chat et al., 2022). This approach is a promising alternative to genotyping using DNA microarrays, as it allows one to obtain information on a larger proportion of genetic variations, increasing the power of association tests and subsequent fine-mapping analyses (Wang Q.S., Huang, 2022). However, despite the decreasing cost of NGS-based technologies, GWAS mainly use high-throughput and relatively cheap DNA microarrays containing hundreds of thousands to millions of common genetic markers, which make it possible to test almost the entire genome for associations with the trait being studied. SNP genotyping using DNA microarrays can contain up to 5 % errors depending on the manufacturer (Lamy et al., 2006; Yang et al., 2011; Guo et al., 2014). However, existing protocols for quality control of the obtained data can significantly reduce the number of errors (on average by 1.7 %) (Zhao et al., 2018). Thus, microarrays allow fairly accurate genotyping of samples even for species with high heterozygosity (i. e., with greater genetic variation than expected at Hardy–Weinberg equilibrium) (Bourke et al., 2018). Moreover, at the end of 2023, the cost of genotyping a sample on a microchip was an order of magnitude lower than the cost of NGS sequencing, which makes it possible to cover a much larger sample size with the same project budget. Their main disadvantage when conducting GWAS is that they do not allow detection of an association between an SNP and a trait if the genetic variant is not represented on the microarray.

Additional difficulties in using DNA microarrays may arise because the information (such as the location of SNPs on the chromosome) used to design the chip is out of date or differs between manufacturers. The above problems can be solved by imputation of genotyping data (Pasaniuc et al., 2012). This approach allows us to increase the density of coverage for the genetic variants studied (total number of markers) and the proportion of common variants when conducting a meta-analysis (combining data from different studies and/or genotyping platforms) (Li Y. et al., 2009).

A replacement for DNA microarrays could be low-coverage WGS (lcWGS), in which random regions of the genome are sequenced (Chat et al., 2022). Research shows that lcWGS significantly outperforms microarrays in marker density, which also allows for a more thorough assessment of associations with less common variants. Such data also require imputation using haplotypes (e. g., from the 1000 Genomes Project) (Auton et al., 2015). The costs of ultra-low coverage WGS (sequencing depth ≤ 0.5x) may be comparable to or lower than those of using DNA microarrays, but its potential as an alternative has not yet been sufficiently assessed (Martin et al., 2021).

DNA sequencing and genotyping solve the task of analyzing genetic information in different ways. As such, sequencing allows you to read entire DNA fragments and is therefore applied to identify rare (minor allele frequency < 0.01 %) and de novo mutations, and is widely used to study the structure of individual genes or genome regions. Genotyping, on the other hand, is a faster and more cost-effective method for analyzing genetic variation, which is particularly useful for large-scale genomic studies involving thousands or even millions of samples. Thus, if the goal of a study is to comprehensively examine the genetic architecture of a trait or disease, sequencing is likely the best approach. However, if the focus of the study is on common genetic variants, or analysis of the population or kinship structure of the sample, then genotyping is often sufficient and more effective (Gresham et al., 2008).

## Imputation of genotyping data

Although sequencing the entire genome of hundreds of thousands of people is not yet feasible, significant progress can be made by identifying only a relatively small number of genetic variants in each person. This type of “incomplete” information is still useful because data on any set of SNPs in a group of people allow inferences to be made about many other unobserved variants in the same people. The approach to accomplish this is called imputation.

Methodology

**Fig. 4.**
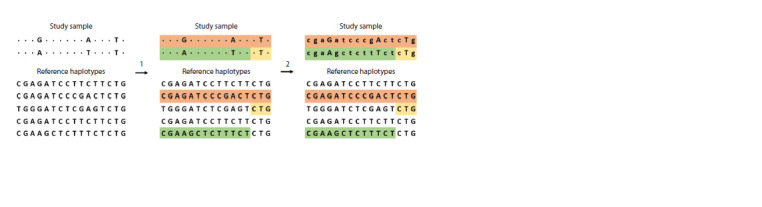
Imputation of genotyping data. 1 – Phasing; 2 – Imputation itself. See the text below for explanations.

The imputation procedure includes the following stages: quality control of genotyping data, phasing, imputation itself, and at the final step – quality control of imputed genotypes (Fig. 4).

Genetic variants that are located nearby on a chromosome are more likely to be inherited together, which occurs because there are only a few recombinations per chromosome. This principle is called “linkage disequilibrium” (LD). Due to this principle, we observe blocks of haplotypes (haploblocks) – sets of closely spaced genetic variants that were inherited together during evolution

In imputation, haploblocks are used to identify common short stretches of DNA on chromosomes that individuals in a randomly selected population may have inherited from a common ancestor. By comparing haplotypes in two samples (study and reference) based on a set of common genetic variants, imputation algorithms provide inferences about the genotypes of the studied individuals. Both of these samples must be from the same ethnic group for imputation to produce accurate results (Mills et al., 2020).

Although genotyping data do not contain haplotype information, it can be inferred and reconstructed using stepwise analysis. Phasing is the process of statistically estimating haplotypes. Imputation can be performed on both raw unphased genotyping data and reconstructed mixed haplotypes, although phasing is known to improve imputation accuracy (Anderson et al., 2010). In addition, phasing is often necessary due to the fact that standard imputation algorithms (more about them below) work specifically with haploblocks

## Quality control of genotyping data

An important step in any genomic study is to conduct data quality control. The importance of this step is illustrated by the example of a paper published in Science that was retracted due to insufficient consideration of technical errors in genotyping on an Illumina chip (Marees et al., 2018).

Quality control of DNA microarray genotyping data is divided into two main steps: control at the individual level and control at the marker level. Individual-level control involves removing a sample in the following cases (Anderson et al., 2010):

– there is an observed discrepancy between the phenotype and the genotype (in particular, the phenotypic sex differs from the genetic one); – the number of heterozygous loci in the genome deviates from the expected value (an overestimation or underestimation of this indicator may indicate sample contamination or inbreeding, respectively); – the sample contains duplicates, relatives of the first or second degree (similar genotypes will be overrepresented, as a result of which allele frequencies in the population may be displayed unreliably); – has a different ethnic origin, that is, there is a stratification of the population (the most common approach for identifying such individuals is principal component analysis (PCA) on a kinship matrix). Data quality control at the level of individual markers also consists of several points that involve the removal of SNPs if: – minor allele frequency (MAF) <0.01; – they are absent from a large part of individuals in the sample; – they deviate significantly from Hardy–Weinberg equilibrium.

To carry out quality control, a number of publicly available programs are used: PLINK 1.9/PLINK 2 (Purcell et al., 2007; Chang et al., 2015), RICOLI (Lam et al., 2020), SMARTPCA (Price et al., 2006) and FlashPCA (Abraham et al., 2017).

## Imputation Tools

**Fig. 5.**
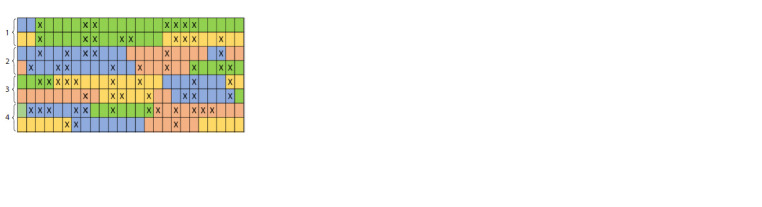
Visualization of the performance of HMM-based algorithms for four individuals from the reference sample. Each column is a separate SNP with two alleles (empty and crossed out squares represent different alleles of the same SNP), and each pair of rows represents two copies of DNA (from each parent). Closely related SNPs are grouped by color, and each haplotype is modeled as a mosaic of color combinations (Scheet, Stephens, 2006).

Over the past twenty years, several different research groups have developed and published a number of tools for phasing and subsequent imputation, most of which are based on the hidden Markov model (HMM) of Li and Stephens (Li N., Stephens, 2003). This statistical model, first described in 2003, assumes that haplotypes are inherited as haploblocks and that recombination events occur at their boundaries. The model probabilistically reconstructs the studied haplotypes in the form of a mosaic composed of haplotypes from a small reference sample (Fig. 5). It has been shown that methods based on Li and Stephens’ HMM are more accurate and efficient (Weale, 2004) than approaches such as Clark’s algorithm (Clark, 1990) or the EM algorithm (Expectation-Maximization) (Dempster et al., 1977) (Browning S.R., Browning B.L., 2011). Currently, the most commonly used programs implementing Li and Stephens’ HMM are Beagle 5 (Browning B.L. et al., 2021), Eagle2 (Loh et al., 2016) and ShapeIT (Delaneau et al., 2012) for phasing, and also Beagle 5 (Browning B.L. et al., 2018), Impute5 (Rubinacci et al., 2020) and Minimac4 (Das et al., 2016) for imputation. Beagle 5 and ShapeIT2 allow you to perform both of these procedures.

A comparative analysis of current phasing and imputation software showed that, overall, Beagle 5.4 performed slightly better than Impute5 and Minimac4, with a higher concordance rate and high performance even on large data sets (De Marino et al., 2022). However, Minimac4 and Impute5 tend to perform better on rare variants because, unlike Beagle 5.4, which computes clusters of haplotypes and performs calculations based on them, Impute5 and Minimac4 search the entire haplotype space. Minimac4 requires the least amount of memory, but calculations take longer. If memory usage is limited and the loss of accuracy is acceptable, then Minimac4 may be the optimal choice of imputation software.

The above programs can be run from a local server and require reference haplotypes. Nevertheless, most of these large-scale datasets are not publicly available. For this reason, special servers that contain information about different reference panels are most often used for imputation of human data, such as Michigan Imputation Server https://imputationserver.sph.umich.edu/index.html#!pages/home (Das et al., 2016) and TOPMed Imputation Server https://imputation.biodatacatalyst.nhlbi.nih.gov/#!pages/home (Das et al., 2016). Researchers can upload their datasets there, configure parameters through the web user interface (select tools, reference panels, etc.), perform phasing and genotype imputation on the server, and download the output files.

As disadvantages of this approach, it is worth noting the need to send your data outside the local server (albeit using secure connection protocols) and possible queues. In addition, users are often limited in the choice of programs or reference panels, and cannot combine multiple panels or integrate their own. However, it is possible to bypass these restrictions, for example, using Docker software (Das et al., 2016), and run imputation on your server. The problem with standalone running is a little more complexity due to manual settings, where the user needs to install additional programs for the pipeline and account for library conflicts

In Supplementary Material 1 compares the tools available on the two servers described above.


Supplementary Materials are available in the online version of the paper: https://vavilov.elpub.ru/jour/manager/files/Suppl_Berd_Engl_28_6.pdf


## Reference panels for imputation of human genotyping data

One important issue in genotype imputation is how to select a reference panel that provides high imputation accuracy in the population of interest. As it was shown (Huang, Tseng, 2014), the quality of imputation is affected not only by the size of the panel, but also by the ethnic composition of the reference sample. The most commonly used panels for European populations currently are 1000 Genomes (Sudmant et al., 2015), Haplotype Reference Consortium (HRC) (Haplotype Reference Consortium, 2016) and Trans-Omics for Precision Medicine (TOPMed) (Taliun et al., 2021).

The 1000 Genomes Phase 3 Version 5 reference panel was prepared as part of the 1000 Genomes Project in 2008 (Auton et al., 2015). In total, while using a combination of low-coverage whole-genome sequencing, high-coverage exome sequencing and microarray genotyping, this project was able to characterize 88 million genetic variants (84.7 million SNPs, 3.6 million short insertions/deletions and 60,000 structural variants). This version of the reference panel includes 49 million markers from 2,504 individuals from a mixed population.

The HRC r1.1 2016 reference panel was compiled by the HRC (The Haplotype Reference Consortium) to create a large haplotype reference panel. The HRC panel combines datasets from 20 different studies, most of which were obtained using low-coverage (4–8x) whole-genome sequencing and consist of samples of individuals of predominantly European ancestry. The reference panel consists of 64,976 haplotypes of 32 thousand individuals with 39,235,157 SNPs; it does not contain deletions or insertions.

The TOPMed (The Trans-Omics for Precision Medicine) project was initiated in 2010 with the goal of collecting and analyzing whole-genome sequencing data. As of September 2021, TOPMed has approximately 180 thousand participants, predominantly of non-European origin, from more than 85 different studies. A reference panel was created based on the TOPMed database, which includes 286,068,980 SNPs; 5,815,513 insertions and 16,222,592 deletions in the genotypes of 97,256 individuals. These genetic variants are distributed across 22 autosomes and the X chromosome. TOPMed (Version r2) is the first panel that is based solely on deep wholegenome sequencing data and is significantly superior to previously published alternatives.

Although most genetic studies and reference panels focus on samples of individuals of European ancestry, it is worth noting that there are various projects aimed at studying the genetic diversity of other populations. These include ChinaMAP (10,588 samples and 136.7 million SNPs) (Li L. et al., 2021), NARD (1,779 individuals, 40.6 million SNPs) (Yoo et al., 2019), GAsP (1,739 samples, 1 million autosomal SNPs) (Wall et al., 2019), SG10K (4,810 samples, 89.1 million SNPs) (Wu et al., 2019) for samples of people of Asian descent, AFAM (2,269 samples, 45 million SNPs) (O’Connell et al., 2021) and UGR (4778 samples, 2.2 million markers) (Fatumo et al., 2022) for African Americans. The TOPMed panel can also be used to impute non-European samples of individuals of both African and Asian descent.

The ideal solution when selecting a panel for imputation is to combine data from multiple reference samples to construct a combined reference panel. However, different studies tend to use different quality control and variant filtering strategies, which can make pooling results difficult.

Another major issue is restrictions on shared data use. For example, individual-level genotype information in many reference panels is not publicly available; therefore, it may not be possible to directly combine it with sequencing results from other samples. In this regard, the meta-imputation method was proposed (Yu et al., 2022). Instead of combining reference panels, genotypes are first imputed using multiple reference panels separately and then the imputed results are combined into a consistent data set.

## Assessment of imputation quality

The quality of genotyping data imputation can be assessed: 1) using standard imputation quality metrics; 2) empirically (for example, conduct a GWAS on the trait of interest and check the reproducibility of association signals known from the literature, or calculate a polygenic estimate of the trait and compare it with real phenotypes).

Imputation quality metrics can also be divided into two large groups (Stahl et al., 2021): 1) those that assess the quality of imputation without using directly genotyped SNPs and are calculated automatically when running the corresponding imputation software, and 2) those that allow the comparison between imputed SNPs and genotypes and are calculated manually.

Quality metrics in the first group are specific to each individual program. For example, for Minimac4 and Beagle 5, the R2 indicator is estimated (Marchini, Howie, 2010), which is calculated differently for each program, while Impute5 calculates the Info parameter (Marchini, Howie, 2010). Because of their specificity, they are not suitable for comparing the quality of data imputed by different methods. This task is successfully accomplished by metrics from the second group, which include: concordation rate (CR), Imputation Quality Score (IQS) (Lin et al., 2010), Hellinger score (Roshyara et al., 2014), squared Euclidean norm score (SEN) (Roshyara et al., 2014) and others. In practice, standard metrics of the first group are most often used.

While conducting imputation, the posterior probabilities of the genotype are estimated. Thus, for biallelic SNPs in an additive model (where the genotype is coded as 0, 1 and 2, and the reference and alternative allele are 0 and 1, respectively), the estimated probability of individual i to have genotype j at a particular locus is denoted as G i j ( j = 0, 1, 2). This indicator is calculated by appropriate imputation software based on data from the reference and target samples using built-in algorithms (for example, a hidden Markov model, as described above). The dose of the alternative allele is calculated as Di = G i1 + 2G i2.

The R2 metric is an approximation of the squared correlation between the imputed allele dose and the expected genotype and is calculated as the ratio of the allele dose dispersion and the expected dispersion under Hardy–Weinberg equilibrium

**Formula. 1.**
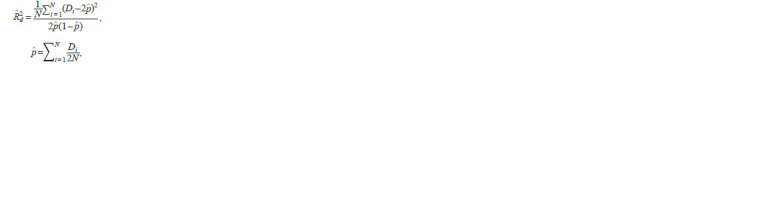
Formula1

where N is the number of individuals in the sample; Di is the dose of the imputed allele for the i-th individual; ^p is the allele frequency estimate

Many modern algorithms (such as Minimac) carry out imputation on pre-phased genotypes, that is, they work with haplotypes. The formula undergoes slight changes, as the set of genotypes is now described as a pool of 2N binary encoded alleles

**Formula. 2.**
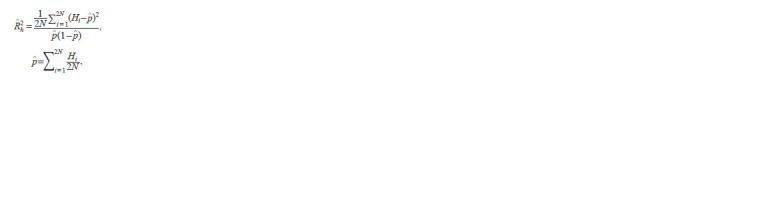
Formula2

where Hi is the probability of the imputed allele in the i-th haplotype (varies from 0 to 1 and is estimated by built-in hidden Markov model algorithms); N is the sample size; ^p is the allele frequency estimate. The derivation of the formulas can be found in Supplementary Materials 2 and 3.

When calculating metrics of the second type, part of the information about genotypes in the sample under study is artificially “masked” (removed from the general data set, while maintaining information about these SNPs). Then the resulting gaps are imputed and compared with real genotypes. For instance, CR represents the proportion of correctly calculated SNPs to all SNPs. The Hellinger exponent is a measure of the distance between two genotype probability distributions and is based on the Bhattacharyya coefficient (Bhattacharyya, 1943), which measures the degree of overlap between two distributions. The SEN metric is the scaled Euclidean distance between the true and imputed dose distributions. Both the Hellinger score and the SEN score are calculated for each individual’s distinct SNPs. IQS is based on Cohen’s kappa statistic and allows for random co-occurrence between imputed and real SNPs.

As mentioned at the beginning, in addition to the listed metrics, a polygenic score (PGS) of the trait can be used to control the quality of imputation (Choi et al., 2020). It is a measure of an individual’s genetic risk for a trait, obtained by summing the quantified effect of many common variants (typically with minor allele frequencies ≥ 1 %) in the genome, each of which may make a small contribution to an individual’s genetic risk for that trait or disease. PGS is typically calculated as a weighted sum of a set of genetic variants, usually SNPs, defined as single base pair variations from a reference genome. The resulting score has a distribution close to normal in the general population, with higher scores indicating higher risk.

In general, the equation for calculating a weighted polygenic risk score for an individual is as follows (Collister et al., 2022):

**Formula. 3.**
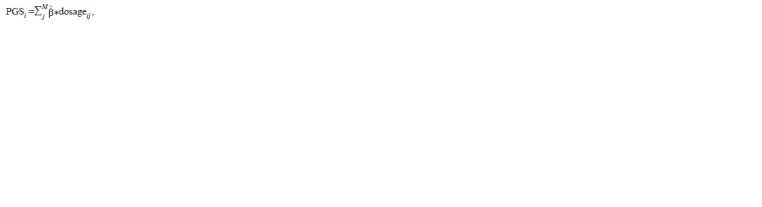
Formula3

where M is the number of SNPs in the model; ^β j is an estimate of the effect size of the j-th variant; dosageij is the genotype encoded 0, 1, 2 for the j-th variant in the genotype of the i-th individual. SNP effect sizes (β) are often obtained from GWAS results.

After calculating the PGS score for a trait, its values are compared with the values of real phenotypes. If there is a significant correlation between these two data sets, we can conclude that the data is of high quality after imputation.

## Examples of imputation in genomic studies on Russian samples

Despite the advantages of imputation and phasing described above, there is very little reference to their use in studies of Russian samples. As such, in a 2023 study on depression in a sample of 4,520 individuals from various regions of Russia, imputation was carried out using the HRC and 1000G reference panels using Beagle 5.1 (Pinakhina et al., 2022). Similarly, in a study of the genetic structure of the Western Russian population (sample of 4,145 individuals), the HRC panel was chosen as the panel for imputation; the procedure itself was carried out using Beagle 4.0 and allowed to consider another 10,454,514 imputed genotyped variants in the analysis, in addition to 623,249 genotyped ones (Usoltsev et al., 2023). And in a 2022 study of markers associated with muscle strength and power in 292 Russians (83 of them professional athletes), not only imputation on a 1000G panel, but also phasing using SHAPEIT was carried out (Moreland et al., 2022).

As stated earlier, one of the most important factors for performing high-quality imputation is the correct choice of reference panel. The authors of one work (Kolosov et al., 2022) assessed the reliability of imputation of genotypes of a sample of 230 elderly people from St. Petersburg (501,100 SNP) by such panels as HRC, 1000G, HGDP (Human Genome Diversity Project (Cann et al., 2002) – a reference panel based on 929 people of various ethnic backgrounds). They were able

to increase the total number of variants studied to 37.6, 37.5 and 26.6 million SNPs for each of the panels, respectively, using Beagle 5.1 (the data were pre-phased). In addition, HRC, compared to the other two panels, showed the highest imputation accuracy (IQS and CR metrics).

All of these works use HRC or 1000G as reference panels, but this approach is somewhat outdated and is subject to revision due to the emergence of a larger TOPMed data set, the use of which serves as a kind of gold standard in international studies at the moment. As for the software, various versions of Beagle are used in the reviewed works.

In the mentioned studies on Russian samples, meta-analyses or fine mapping of genes were not carried out; however, as examples from other works show (Barton et al., 2021), thanks to imputation and phasing such analyzes can be done with a significant quality improvement.

## Conclusion

From the above, we can conclude that, at the moment, imputation of genotyping data is an integral part of many human genomic studies, in particular GWAS. It provides an increase in the number of SNPs analyzed and makes it possible to combine the results of different studies. Imputation also significantly improves the results of fine mapping, allowing the most accurate identification of specific genetic variants and genes that determine the association of the entire genome region with the trait being studied (Chundru et al., 2019).

It is worth noting that for large-scale studies where sample size and genotyping coverage are important, the combination of DNA microarrays/sequencing with low coverage and further imputation is the most optimal and cheapest data acquisition strategy suitable for most genomic study designs. This combination is used in all major national biobanks, such as UK Biobank (Sudlow et al., 2015), AllOfUs (Ramirez et al., 2022) and others

Along with the listed advantages, the imputation method has a number of disadvantages and limitations. In particular, reading errors due to low coverage, as well as incorrect selection of parameters for imputation along with an inappropriate reference panel, often lead to low accuracy of the imputed data, which can negatively affect the results of further stages of analysis. It must also be remembered that imputation uses information about haplotypes from the reference sample, so when it becomes outdated, genetic variants that have become frequent in the population relatively recently may be imputed worse (Ali et al., 2022). In addition, a high level of recombination reduces the accuracy of phasing and subsequent imputation of genotypes, and therefore, in some cases, additional recombination analysis is necessary (Weng et al., 2014).

Also, imputation can smooth out genetic differences between individuals in case-control samples (Lau et al., 2024): imputed data may introduce inaccurate genotypes in regions where differences between case and control are expected, and this effect appears regardless of how large and diverse the reference panel is. Finally, when using the method, it is important to remember that what is true for the population as a whole may not always be true for a specific individual.

Currently, there is a wide variety of programs and reference panels for imputation of human genomic data, and, as a consequence, many combinations of them. Due to this, researchers have the opportunity to select the optimal set of imputation tools for the characteristics of the sample and the objectives of a particular study. A review of works on Russian samples showed that the most popular software for imputation is Beagle of various versions, and among reference panels, HRC and 1000G are most often used, which is somewhat different from international practices, where the leader among reference panels is TOPMed.

Greater awareness of the intricacies of imputation and a deliberate approach to the selection of tools will improve the quality of genomic data without increasing the cost of obtaining them, facilitate their integration with the results of other studies, and provide more accurate information about the genetic control of human traits.

## Conflict of interest

The authors declare no conflict of interest.
